# Pakistan’s sudden HIV surge: a national crisis with international implications

**DOI:** 10.1097/MS9.0000000000004239

**Published:** 2025-10-30

**Authors:** Mir Raza Ali, Abdul Khalique, Noor un nisa Irshad, Sakan Binte Imran

**Affiliations:** a Department of Medicine, Jinnah Postgraduate Medical Centre, Karachi, Pakistan; b Department of Medicine, Jinnah Sindh Medical University, Karachi, Pakistan; c Department of Medicine, Sir Salimullah Medical College, Dhaka, Bangladesh

*To the Editor*,

The recent surge in human immunodeficiency virus (HIV) incidence in Pakistan has sparked widespread alarm, both domestically and internationally. According to the National AIDS Control Programme, Pakistan reported approximately 9700 new HIV infections in the first 9 months of 2024, making it one of the fastest-growing HIV rates in South Asia^[1]^. This rapid growth reflects long-standing inadequacies in awareness, prevention, and healthcare infrastructure, which now threaten to spread throughout global health systems^[2]^.

This crisis has many underlying causes. Unregulated medical practices, unsafe blood transfusions, and syringe reuse continue to be significant factors, particularly in rural clinics^[3]^. Practices are still the same despite a significant outbreak in Sindh in 2019, where hundreds of youngsters caught HIV from reusing syringes^[3,4]^. Furthermore, open communication, prompt testing, and treatment are impeded by the low literacy rate and pervasive stigma associated with HIV^[4]^.

Social and behavioral aspects are also very important. HIV has been able to spread from high-risk groups to the general community due to unsafe sexual behaviors, injecting drug use, and inadequate access to harm-reduction interventions^[2,5]^. Only over 16% of Pakistani HIV-positive individuals receive antiretroviral therapy (ART), according to the Joint United Nations Programme on HIV/AIDS (UNAIDS), leaving a sizable untreated population that contributes to continued transmission^[5]^. The problem is made worse by a lack of thorough sex education and insufficient support for healthcare^[4,6]^.

It is impossible to ignore Pakistan’s HIV epidemic’s worldwide ramifications. Pakistan is home to a sizable population of refugees and migratory workers who regularly traverse international borders, increasing the danger of transmission to nearby nations and beyond^[1,5]^. Additionally, this increase jeopardizes the UNAIDS 95-95-95 strategy’s global objective of eliminating AIDS (acquired immunodeficiency syndrome) as a public health problem by 2030^[6]^. Furthermore, because of the increasing prevalence, Pakistan needs international cooperation in the form of finance, technical help, and information sharing^[2,6]^.

Reversing this trend requires immediate action. It is crucial to improve access to ART, expand HIV testing, ensure safe injection and transfusion procedures, and strengthen infection control^[3,5]^. Public education initiatives need to raise awareness and fight stigma, particularly in rural areas where myths are still prevalent^[4]^. Finally, since unchecked expansion in one nation puts everyone else at risk, Pakistan’s solution needs international support^[1,6]^. This article aligns with the TITAN Guidelines on the need for transparency in AI use in healthcare^[7]^.

In conclusion, the unexpected rise in HIV cases in Pakistan is a global warning rather than just a domestic health issue. Future generations can be shielded from an avoidable epidemic by a coordinated, evidence-based, and caring response.

## Data Availability

Not applicable.
